# Demographic Structure, Sex Ratio and Growth Rates of Southern Bluefin Tuna (*Thunnus maccoyii*) on the Spawning Ground

**DOI:** 10.1371/journal.pone.0096392

**Published:** 2014-05-05

**Authors:** Jessica H. Farley, J. Paige Eveson, Tim L. O. Davis, Retno Andamari, Craig H. Proctor, Budi Nugraha, Campbell R. Davies

**Affiliations:** 1 Wealth from Oceans Flagship, CSIRO Marine and Atmospheric Research, Hobart, Tasmania, Australia; 2 Institute for Mariculture Research and Development, Gondol, Bali, Indonesia; 3 Research Institute for Tuna Fisheries, Denpasar, Bali, Indonesia; Texas A&M University, United States of America

## Abstract

The demographics of the southern bluefin tuna (SBT) *Thunnus maccoyii* spawning stock were examined through a large-scale monitoring program of the Indonesian longline catch on the spawning ground between 1995 and 2012. The size and age structure of the spawning population has undergone significant changes since monitoring began. There has been a reduction in the relative abundance of larger/older SBT in the catch since the early 2000s, and a corresponding decrease in mean length and age, but there was no evidence of a significant truncation of the age distribution. Pulses of young SBT appear in the catches in the early- and mid-2000s and may be the first evidence of increased recruitment into the spawning stock since 1995. Fish in these two recruitment pulses were spawned around 1991 and 1997. Size-related variations in sex ratio were also observed with female bias for fish less than 170 cm *FL* and male bias for fish greater than 170 cm *FL*. This trend of increasing proportion of males with size above 170 cm *FL* is likely to be related to sexual dimorphism in growth rates as male length-at-age is greater than that for females after age 10 years. Mean length-at-age of fish aged 8–10 years was greater for both males and females on the spawning ground than off the spawning ground, suggesting that size may be the dominant factor determining timing of maturation in SBT. In addition to these direct results, the data and samples from this program have been central to the assessment and management of this internationally harvested stock.

## Introduction

High fishing pressure can affect the size and age structure of a fish population, often resulting in a reduction in the relative abundance of larger and, indirectly, older individuals (age-class truncation) [1]. In stock assessments, it is often assumed that spawning stock biomass (SSB) represents the stock' reproductive potential, ignoring changes that may have occurred in the size and age structure of the spawning population. Monitoring changes in the size and age composition of the spawning population, as well as collecting reproductive data to estimate potential annual fecundity, is central to quantifying the impacts of fishing on total egg production. Directly estimating the age structure is particularly important for long-lived species where fish size is unlikely be a good predictor of age. Changes in the demographics of a spawning stock, such as mean length or age and sex ratio, can also be used as indicators of the status of the stock for management purposes.

Southern bluefin tuna (SBT) *Thunnus maccoyii* is a long-lived, late-maturing tuna reaching a maximum size and age of at least 200 cm and 40 years respectively [2]. SBT has been harvested commercially since the early 1950s and is currently estimated to be at a small fraction of its original spawning stock size [3].The spawning ground for SBT is located in the north-east Indian Ocean between Indonesia and the north-west coast of Australia. Mature fish migrate to this region from the Southern Ocean and Tasman Sea to spawn between September and April each year [4], [5], [6]. An Indonesian longline fishery targeting yellowfin (*Thunnus albacores*) and bigeye tuna (*Thunnus obesus*), with a bycatch of SBT, has operated on the spawning ground since the late-1970s (Table 1) [7], [3]. The catch of SBT by Indonesia increased rapidly from just a few tonnes to 2,500 tonnes by 1999 and then declined to <650 tonnes in 2003 and 2004. In October 2005, the price of fuel in Indonesia doubled after the Indonesian Government cut fuel subsidies. The catch of SBT spiked just prior to the price increase as vessels quickly returned to port, but the price rise had a significant impact on fishing operations with less longline activity and fewer SBT landings [8]. Vessels that continued to operate stayed at sea for much longer periods (2–5 months) and used fishing vessels as carriers to bring fish back to port [8], [9]. Since joining the Commission for the Conservation of Southern Bluefin Tuna (CCSBT) in 2008, Indonesia has been allocated a national total allowable catch (TAC) ranging between 651 to 750 tonnes per year.

**Table 1:** Estimated total annual landings (tonnes) of tuna at Benoa, Bali, by species since 1993.

Year	Southern bluefin	Yellowfin	Bigeye	Albacore
1993	1191	14596	6192	716
1994	786	10815	5360	338
1995	721	10590	6543	463
1996	1404	11061	10536	1035
1997	1922	12047	10115	2372
1998	1151	15735	12611	905
1999	2178	16128	9945	1687
2000	1046	12596	8676	2238
2001	1419	12165	9362	2461
2002	1631	10380	11646	2257
2003	556	7399	5611	3408
2004	613	4413	4184	1906
2005	1690	4196	3939	1494
2006	558	4323	4366	1450
2007	1077	5354	5292	1132
2008	905	6924	5033	2811
2009	746	7240	4680	1020
2010	566	5372	2168	983
2011	432	3006	2504	384
Total	20592	174340	128763	29060

Data sourced from Andamari et al. (2004), Proctor et al. (2011), and Satria et al. (2012).

Given the significant decline in the SSB and average recruitment of SBT [3], it was important to obtain accurate estimates of the size and age distribution of SBT landed by the Indonesian longline fishery for monitoring changes in the spawning population over time and the impact of management measures. In 1992, a catch monitoring program was established at Benoa (Bali) to examine the catch composition of tunas landed by Indonesia [7], [10]. The program focussed on the collection of size data of SBT caught by the longline fishery as well as biological samples, such as otoliths and ovaries, for analysis of age and reproductive dynamics [4], [11], [12]. There were concerns, however, that the SBT data from the Indonesian fishery may not be representative of the spawning population, given that fish caught by Indonesia were generally larger than those caught historically by Japan on the spawning ground [13]. Subsequent work found that SBT segregate by size on the spawning ground, and that this size partitioning was related to spawning activity [11]. It appears that large fish spend proportionally more time spawning while on the spawning ground than small fish, and since spawning occurs at the surface, larger fish are more likely to be caught in shallow (Indonesian) longline sets, and smaller fish are more likely to be caught in deep (Japanese) longline sets. This suggests that, in lieu of survey data from which a more complete estimate of the size distribution on the spawning ground could be derived, SBT caught in shallow longline sets may better represent the spawning population.

This long-term monitoring of the Indonesian fishery and collection of large quantities of high quality length and direct age data has allowed us to estimate the size and age distribution of the Indonesian catch over the past two decades, examine changes in size/age based parameters over time, and estimate sex ratio and sex specific growth rates of the spawning population. It has also allowed us to examine whether there have been changes in fishing practices, such as depth of fishing of the Indonesian fleet, which may assist in explaining the changes in the size/age structure of the spawning population.

## Methods

### Ethics statement

Ethical approval was not required for this study, as all fish were collected as part of routine fishing procedures. No samples were collected by the authors. All samples in this study originated from the Indonesian longline fishery and were already dead when sampled as part of commercial processing operations. Fish were sacrificed by the commercial fisher at sea using standard fisheries practices. Permission was granted to use samples from all fish. All samples were donated. No field permits were required to collect samples, since all originated from commercial catch. SBT are not a protected species in any ocean.

### Catch monitoring

Catch data were obtained from Indonesian longline landings through the Benoa-based monitoring program for the 1995 to 2012 spawning seasons (a spawning season is defined as July 1 of the previous year to June 30 of the given year). The data were collected through a series of collaborative research programs between Australia's CSIRO Marine and Atmospheric Research, Indonesia's Research Centre for Capture Fisheries and Research Institute for Marine Fisheries, the Indian Ocean Tuna Commission, and Japan's Overseas Fishery Cooperation Foundation [8]. In mid-2002, the monitoring program expanded to include the ports of Muara Baru (Jakarta) and Cilacap (south coast Central Java); however, the majority of targeted SBT sampling still occured at Benoa as this is the port where the majority (85%) of SBT is landed [14]. Landed SBT are graded into export and non-export quality based on flesh quality. Quality and grading is dependent on handling, length of trip and/or condition of fish at capture, rather than fish size [11]. Generally, only SBT graded as not suitable for export were available for monitoring.

Fork length (*FL*) was measured to the nearest cm (Table 2) and length frequencies were constructed for each spawning season. Through the monitoring program, one fishing company was identified as having vessels operating well south of the spawning ground (southern zone) from January 2004 to April 2007. Since it is unknown if fish caught in the southern zone were mature or would migrate to the spawning ground, it was important that these fish were identified in the size data and excluded from our analysis so that the estimated size distribution of the spawning population compared over time was consistent. The size distribution of SBT landed by this company (Company A) was compared to the other companies (combined data) each season using the two-sample Kolmogorov–Smirnov (K-S) test to determine if significant differences were present.

**Table 2:** Number of SBT samples by fork length (*FL*), dressed weight (*DW*), sex, otoliths and age spawning season.

Spawning season	*FL* (cm)	*DW* (kg)	Sex	Otoliths	Age	%F	Mean length (cm)	Mean age (years)	Age diversity index	Mean BE index
1995	1610	1610		549	486		180.7	21.2	18.3	1.49
1996	1107	1107		225	50		178.9			2.30
1997	1615	1615		602	475		179.6	20.8	20.7	2.53
1998	1577	1577		519	485		176.4	19.8	23.2	2.60
1999	936	936		660	474		179.9	20.7	22.4	1.69
2000	786	786		533	498		177.4	19.5	23.1	1.93
2001	762	762		720	481		174.2	16.9	21.1	2.07
2002	821	821		715	489		169.5	14.8	17.6	1.91
2003	1385	1384		1385	488		166.8	14.5	15.1	2.24
2004	1279	1279		1279	494		168.5	15.2	15.9	1.56
2005	1580	1573		1523	493		170.1	15.3	16.7	2.58
2006	1182	1182		1180	486		169.2	14.4	16.0	2.12
2007	1586	1586		1586	491		168.3	15.1	16.0	2.03
2008	1693	1693		1693	485		169.5	16.7	16.9	1.76
2009	1704	1704	1444	1697	479	53.3*	171.0	15.6	16.5	
2010	1583	1583	1333	1583	488	53.5*	168.5	15.3	16.0	
2011	1015	1015	924	1015	481	53.5*	170.4	16.8	16.2	
2012	565	565	547	543	0	53.9	169.4			
Total	22786	22778	4248	18007	7823		172.4	17.0		2.11

Estimates of percent female (%F), mean length, mean age, age diversity (Shannon-Weaver) index, and bigeye (BE) index are shown for samples from the spawning ground only.

Samples caught south of the spawning ground have been excluded (see text for details). * Indicates significant χ2-tests (P<0.05) (i.e., % females significantly different than 50%).

### Size distribution

To examine changes in the size structure of the spawning population over time, it was necessary to determine whether changes had occurred in the fishing practices of the Indonesian fleet, such as the depth of the longline sets or distribution of effort through the spawning season, which may influence proportion of the population available to be caught and the size of fish caught. A continuous, consistent time series of data on fishing depth are not available for the Indonesian longline fishery. As an alternative we used the catch data obtained through the monitoring program to calculate the relative proportion of bigeye tuna in the landings, referred to as the bigeye (BE) index [11]: BE index  =  weight of bigeye/(weight of bigeye + yellowfin).

The index is based on the assumption/observation that bigeye is generally caught deeper than yellowfin. The BE index was calculated for individual landings from 1995–2009, and was available for 96.8% of the landings in those years which had SBT length measurements. The BE index ranges from 0 to 1 and is divided into 5 levels (0.0–0.2, 0.2–0.4, etc.) for analysis. If the BE index is high, the fishing depth is assumed to be deep (and vice versa). To take into account possible differences in the size distribution of SBT at different depth levels (i.e., different levels of the BE index), we calculated the weighted mean length of SBT caught each spawning season using weights equal to the proportion of fish at each BE index level. Similarly, to examine the effect of month of capture on the size distribution of the catch, we calculated the weighted mean length of SBT caught each spawning season using weights equal to the proportion of fish caught in each month. The only months that had length data collected in all seasons were September to March, thus length data for April to August were excluded (0.3% of length measurements). A comparison was then made with the mean fish length by spawning season calculated using the original sample data (i.e., the unweighted means).

To formalize the above investigations, generalized linear models (GLMs) were fit to the data, with the proportion of small SBT (<165 cm *FL*) as the binomial response and spawning season, BE index and month as potential explanatory variables. The variables and interactions terms to include in the final model were determined using χ^2^ significance tests. Using the best fitting model, the expected proportion of small SBT in each season was estimated for fixed levels of month and BE index.

### Age estimation

Sagittal otoliths were removed from approximately 500 to 1700 SBT measured for length each spawning season (1995–2011), with the exception of the 1996 season when only 225 otoliths were sampled (Table 2). Five hundred were selected from each season for annual age estimation apart from 1996 when only 50 were selected. The number selected was based on the work by Morton and Bravington [15] who estimated that 500 samples would be sufficient to provide acceptable precision; i.e., coefficients of variation (CVs) under 20% for the Indonesian fishery. For the 1995 to 1999 seasons, otoliths were selected randomly from those sampled (which included most otoliths collected). For the remaining spawning seasons, a fixed number of otoliths were chosen from each 1 cm length class to obtain as many age estimates from length classes where sample sizes were small.

All otoliths were prepared and read following the techniques described by Clear et al. [16] and Anonymous [17] and the precision was examined by calculating the CV of replicates readings [18]. To determine the age structure of the Indonesian catch, age-length keys (ALK) were developed using the sample of aged fish for each spawning season except for the 1996 season as too few otoliths were read to develop a reliable ALK. The ALK gives the proportion of fish at age in each 5-cm length class, which is then used to estimate the age-frequency distribution of the catch from the length-frequency distribution obtained through the monitoring. The Shannon-Weaver index was calculated as a measure of age structure diversity, using:
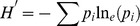
where *p_i_* is the proportion of fish belonging to the *i*th age class [19].

### Sex ratio

Sex was recorded for the majority of fish measured for length for the 2000–2012 spawning seasons. For most fish, sex was determined from a small sample of remnant gonad (RG) tissue left in the visceral cavity when the fish was landed, as SBT lack external sexual characteristics and almost all were landed in gutted form. A secondary method, based on the size and shape of the anus, was used to identify sex when RG tissue was not present. The method used to determine sex was recorded only for the 2009–2012 seasons, thus it was unknown whether the RG or ‘anus’ method was used in the earlier seasons. Exploratory data analysis suggested that the ‘anus’ method was not accurately identifying the sex of all fish, thus only RG data for 2009–2012 were used here (Table 2). The sex ratio of SBT was calculated, and chi square tests were used to examine differences from an expected 1∶1 by season and length class (5-cm).

### Sex-specific growth rates

The distribution of size-at-age between males and females on the spawning grounds were examined using samples for which sex was determined by the RG method (from seasons 2009–2012). A von Bertalanffy (VB) growth curve was fit to the age and length data for each sex separately. Although SBT growth appears to follow a two-stanza VB model, the transition between growth stanzas occurs before age 5 [20]. Since the age of transition is younger than any data from the spawning ground, fitting a simple VB model was considered the most parsimonious approach.

In addition, age and length data for SBT caught off the spawning ground (see [12]) were used to compare length-at-age of SBT on and off the spawning ground. Only samples with sex information were used so that males and females could be compared separately. For all fish caught off the spawning ground, sex was determined by trained observers onboard vessels based on the appearance of the whole gonad [12].

## Results

### Length distribution

**Figure 1:**
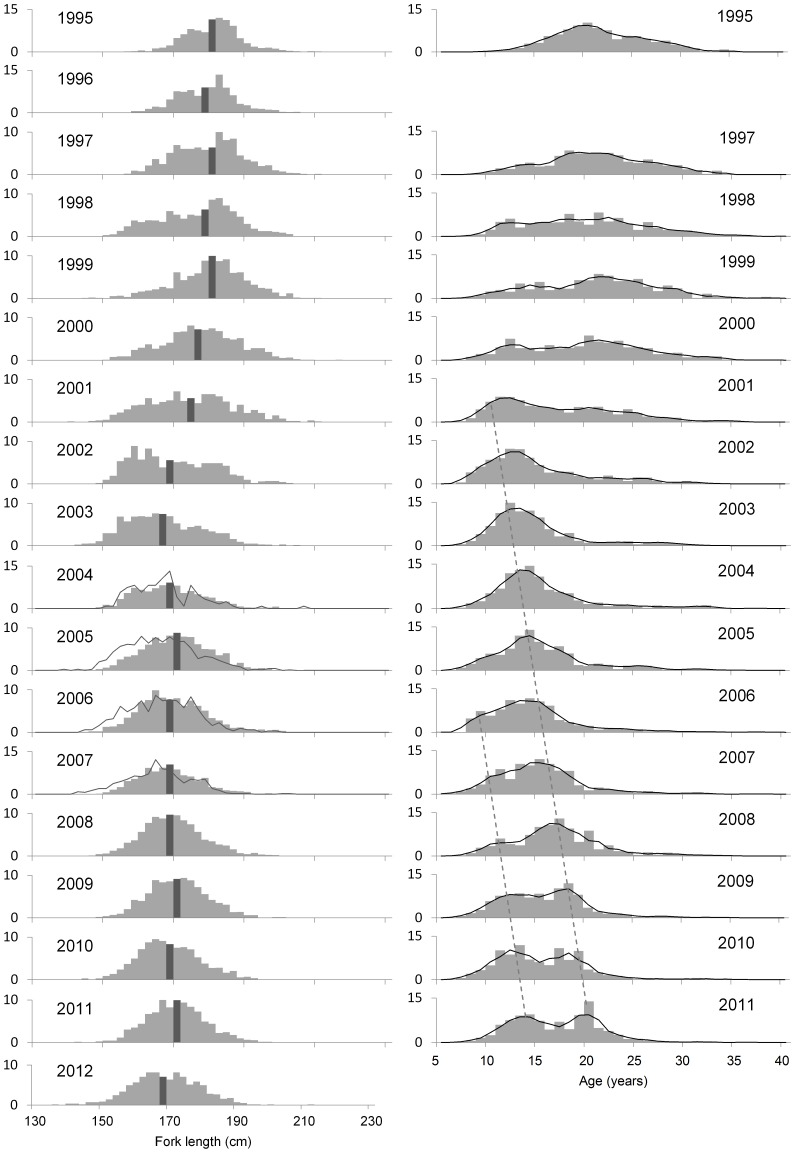
Length (left) and age (right) distribution of the SBT spawning stock by spawning season. The dark grey bar shows the median length class. For comparison, the length distribution of SBT thought to be caught south of the spawning ground (Company A) is shown for the 2004 (n = 121), 2005 (n = 685), 2006 (n = 311) and 2007 (n = 452) seasons (solid grey line). The 3-year running mean of the age distribution is shown (solid black line) and the two possible pulses of recruitment to the fishery (dashed grey line).

Length measurements were obtained for nearly 23,000 SBT from 18 spawning seasons (Table 2). Length data from the landed catch of Company A, identified as having fishing vessels operating in the southern zone, was significantly different to the other companies in the 2005 to 2007 seasons (K-S test; *P*<0.001). The catch by Company A clearly comprised a greater proportion of small fish (140–160 cm *FL*) in each of the seasons in question (Fig. 1). The catch by Company A in the 2004 season also appeared to contain more small fish relative to the other companies, but the difference was not significant (K-S test; *P* = 0.053). Since it was unknown if small SBT caught in the southern zone would migrate to the spawning ground, SBT landed by Company A in the 2004 to 2007 seasons were excluded from further analyses.

**Figure 2:**
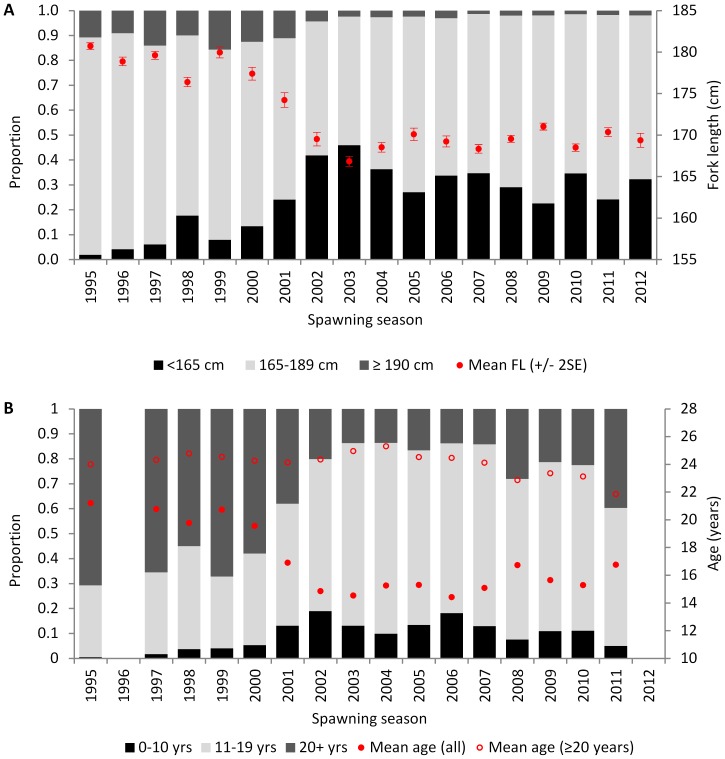
Proportion of SBT caught by size (A) and age (B) classes by spawning season. Mean fork length (*FL*) ± 2SE (upper graph) and mean age of all and SBT ≥20 years (yrs) (lower graph) by spawning seasons are shown in red. Data from Company A operating south of the spawning ground were excluded.

Considerable change has occurred in the size distribution of SBT caught by Indonesian longliners on the spawning ground since monitoring began. In the mid- to late-1990s, the majority of fish caught were between 165 and 190 cm *FL* with a median length of approximately 180 cm *FL* (Fig. 1). In 1998, an increase in the relative proportion of small fish (<165 cm *FL*) occurred but this did not persist into the 1999 season (Fig. 2A). In 2000, the relative proportion of small fish increased again compared to the previous season, and continued to increase steadily, peaking at 45.9% of the catch in 2003 (Fig. 2A). Since then, the relative abundance of fish <165 cm *FL* has fluctuated between 22.5 and 36.9% with a median length of approximately 170 cm *FL*. The mean length of SBT in the catch decreased between 1995 and 2003 from 180.7 cm to 166.8 cm *FL*, and has remained between 168.3 and 171.0 cm *FL* since that time (Table 2; Fig. 2A).

**Figure 3:**
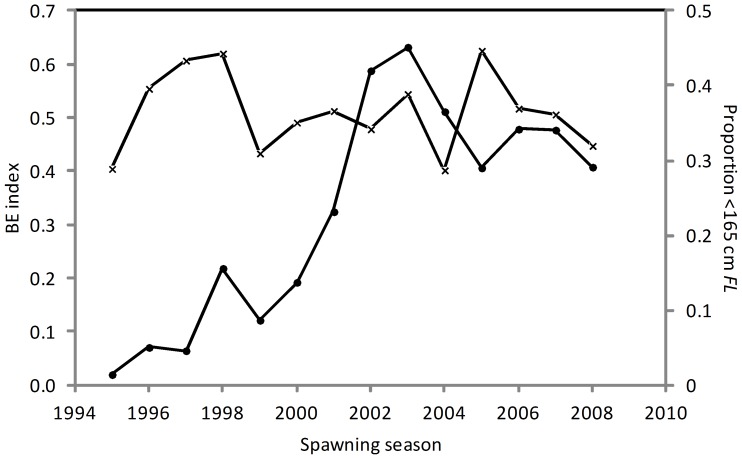
Mean bigeye index (x) and proportion <165 cm FL (•) by spawning season.

**Figure 4:**
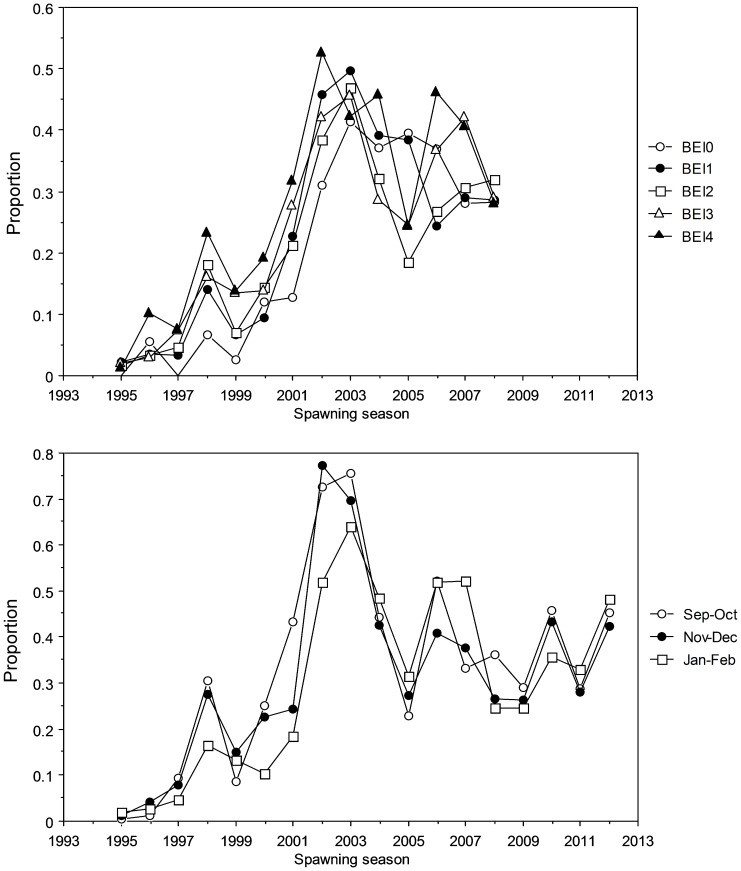
Proportion of SBT <165 cm by spawning season for bigeye index (A) and month-group (B). BEI0 is shallow, BEI4 is deep. BEI  =  bigeye index.

**Figure 5:**
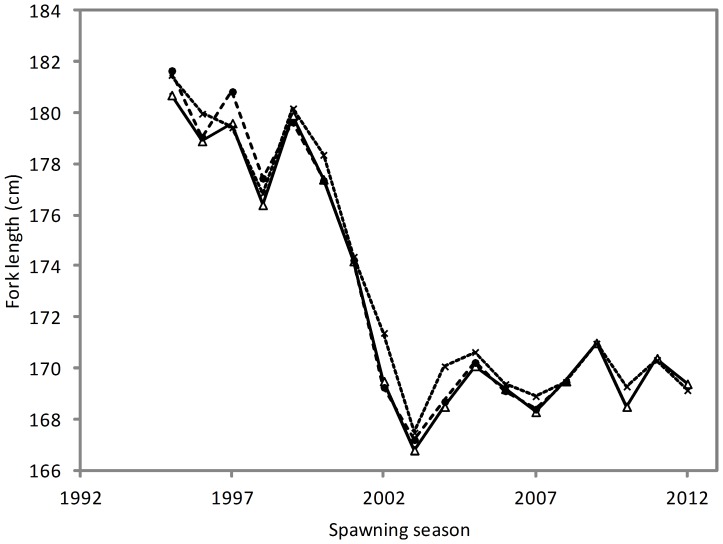
Mean fork length of SBT by spawning season. Data are shown for observed (Δ) and estimated mean length when BE index (•) and month (x) is accounted for in the length distribution of fish caught.

Initial examination of mean BE index by spawning season suggests that there has been no systematic change in fishing depth by the Indonesian fishery that would account for the increase in the relative abundance of small SBT in the 2000s (Table 2). Although there appears to be a relationship between mean BE index (fishing depth) and the proportion of fish <165 cm *FL* in the catch between 1995 and 1999 (Fig. 3), this relationship was not present in the latter years when the greatest change in the size of fish caught occurred. The increase in the proportion of fish <165 cm *FL* in the catch from 2000 occurred at all levels of the BE index and month groups (Fig. 4) suggesting that it occurred independently of changes in fishing practices. This is supported by the comparison of estimated mean length by spawning season when BE index and month of capture were accounted for (Fig. 5). The similarity of the observed and estimated weighted mean length of fish by season suggests that the observed changes in fishing practices had only a minor influence on the size of fish caught.

**Figure 6:**
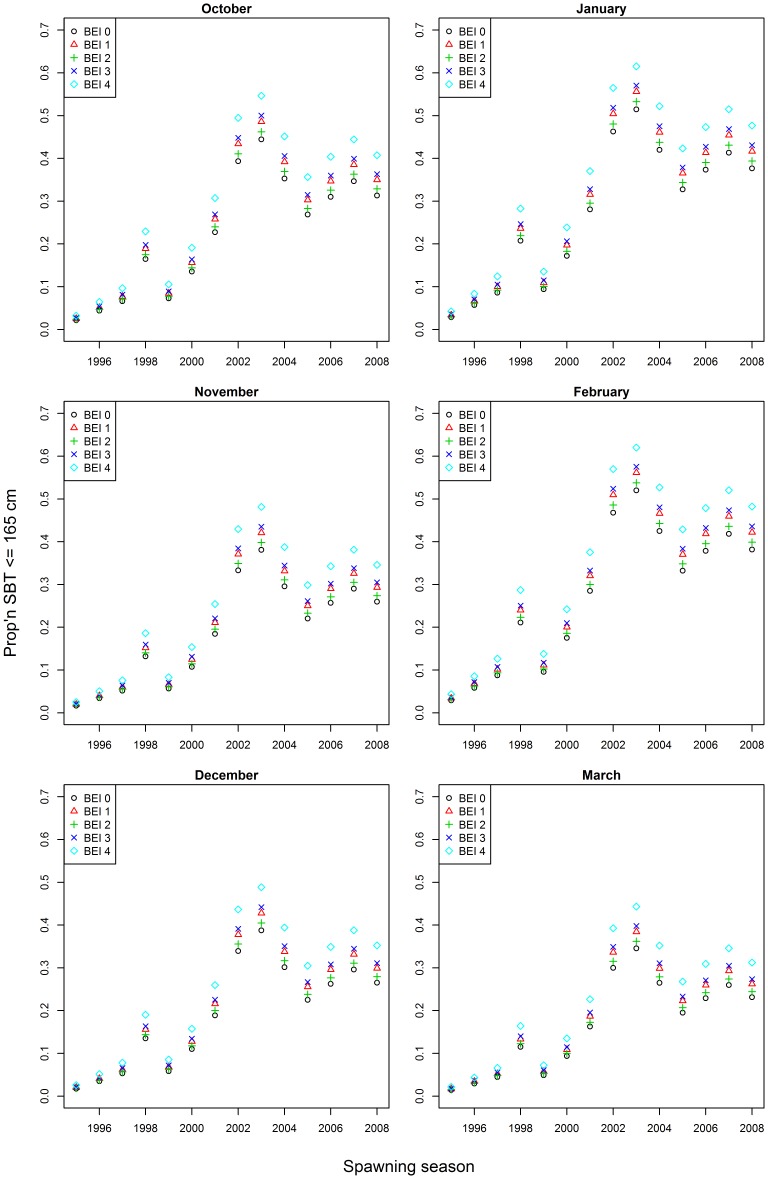
Expected proportion of small SBT each month and level of the bigeye index. Proportions calculated from the best-fitting generalized linear model. BEI0 is shallow, BEI4 is deep. BEI  =  bigeye index.

Results from the GLMs suggest that spawning season, month and BE index are all highly significant explanatory variables of the proportion of small SBT, as are the pair-wise interaction terms between spawning season and month and spawning season and BE index (Table 3). The interaction term between month and BE index did not significantly improve the model fit (χ^2^ test, *P* = 0.235), so was not included in the final model. Using the final model to estimate the expected proportion of small SBT at each level of month and BE index confirms that the proportion of small SBT on the spawning ground increased between seasons 2000 and 2003 (Fig. 6). Although the size of this increase varied among some months and levels of the BE index it was consistently present.

**Table 3:** Analysis of deviance table from fitting a generalized linear model to the proportion of small (≤165 cm) SBT on the spawning ground.

	Df	Deviance	Resid. Df	Resid. Dev	P(>|Chi|)
NULL			15814	21923	
Season	14	6388.8	15800	15534	0.000
Month	6	154.9	15794	15379	0.000
BE Index	4	37.6	15790	15342	0.000
Season:Month	78	302.9	15712	15039	0.000
Season:BE Index	52	133.8	15660	14905	0.000
Month:BE Index	24	28.6	15636	14876	0.235

Results are for the model including spawning season, month and bigeye index as main effects and all pair-wise interactions.

### Age distribution

Age was estimated for 7,773 of the 8,000 SBT selected for age estimation. These fish ranged in size from 122 to 229 cm *FL* and 5 to 40 years. The CV between readings by the primary reader ranged from 3.5–5.3% by spawning season with an overall CV of 4.15%. The second age estimate of the primary reader agreed with the original estimate in 37.6% of cases, and was within two years of the original in 91.7% of cases. The CV between primary and secondary readers was 5.08%. These low levels of error, especially between the two readers, suggest consistent interpretation of age in blind tests.

The minimum age of SBT sampled was 5 years for two unsexed fish, while the maximum age for males and females was 38 years and 40 years, respectively. As expected, the estimated age structure of fish caught by the Indonesian fleet on the spawning ground shows substantial changes over time (Fig. 1; Fig. 2B). Between 1995 and 2000, the majority of fish landed were relatively old (≥20 years) and the mean age was 19–21 years (Fig. 2B; Table 2). After 2000, the mean age of fish decreased to 14–17 years as the abundance of young fish increased in the catch relative to old fish (Fig. 1; Table 2). The age structure diversity index was significantly higher (ANOVA, *F* = 184.5, *P*<0.001) in the 1998–2000 period (mean 22.08±1.3) compared to the 2003–2011 period (mean 16.3±0.55). A clear increase in the relative abundance of young fish occurred in the early 2000s and again in ∼2006, and these modes appeared to progress through the fishery on an annual time step (Fig. 1). The first pulse has a clear mode in 2003 (age 12 years) which follows through to 2011 (age 20 years). These fish would have been spawned in 1991. Similarly, the second pulse has a clear mode in 2006 (age 9 years) which follows through to 2011 (age 14 years). These fish would have been spawned in 1997. The average age of SBT greater than 20 years old has remained relatively stable since monitoring began, although there is some indication of a slight decline in the most recent four or five spawning seasons (Fig. 2B).

### Sex ratio

**Figure 7:**
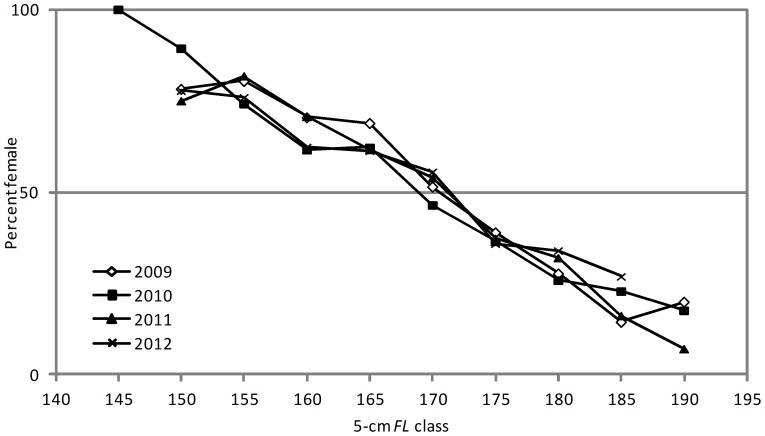
Percent female by 5-cm length class for SBT in the Indonesian catch in 2009–2012. Data point excluded if n<10.

The sex ratio of SBT in the landed catches was significantly different from the expected 1∶1 in 2009 to 2011 with a slight dominance of females (χ^2^ tests, *P*<0.01) (Table 2). Although the percent females was slightly higher in 2012 than in 2009 to 2011, the sex ratio was not significantly different from 1∶1 in 2012 due to the smaller sample size (χ^2^ test, *P* = 0.066). The sex ratio varied with fish length in all seasons, being significantly female-biased in length classes below 170 cm *FL*, and significantly male-biased in length classes above 170 cm *FL* (χ^2^ tests, *P*<0.01) (Fig. 7). Chi-square tests did not indicate significant deviations from a 1∶1 ratio in the 170 cm length class in any season. A clear trend was evident in the percent female with fish length, decreasing steadily from 74–89% in the 150 and 155 cm length classes to only 7–27% in the 185 and 190 cm length classes.

### Sex specific growth rates

**Figure 8:**
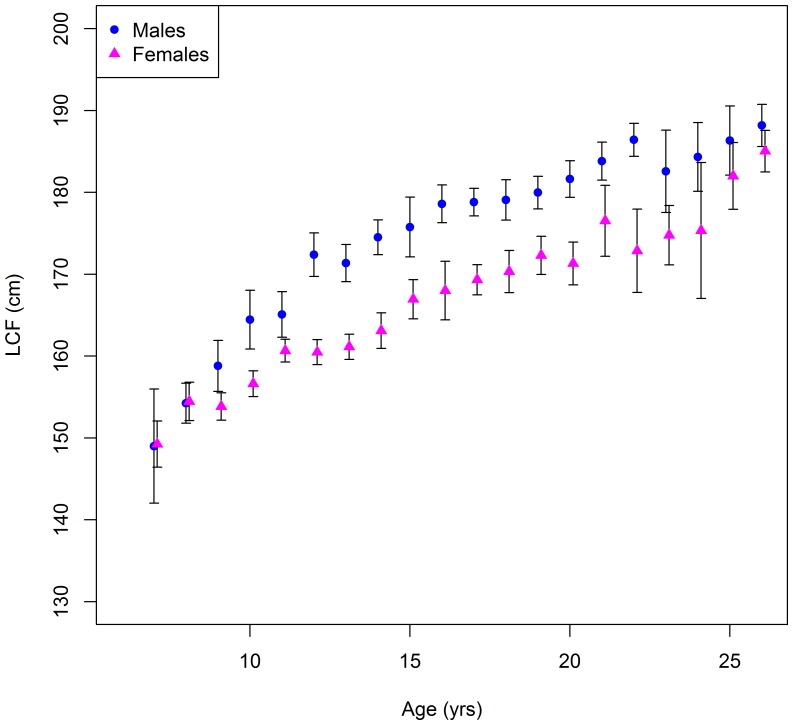
Mean length-at-age ± 2SE for male and female SBT caught on the spawning ground. Note that fish over age 25 were pooled into age group 26+.

**Figure 9:**
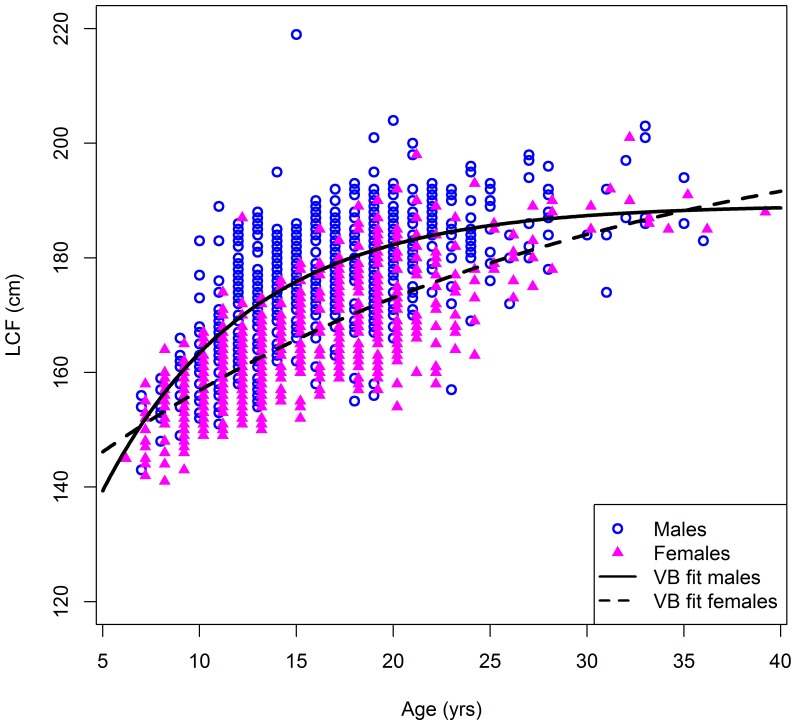
von Bertalanffy (VB) growth curves fitted to the age and length data by sex.

**Figure 10:**
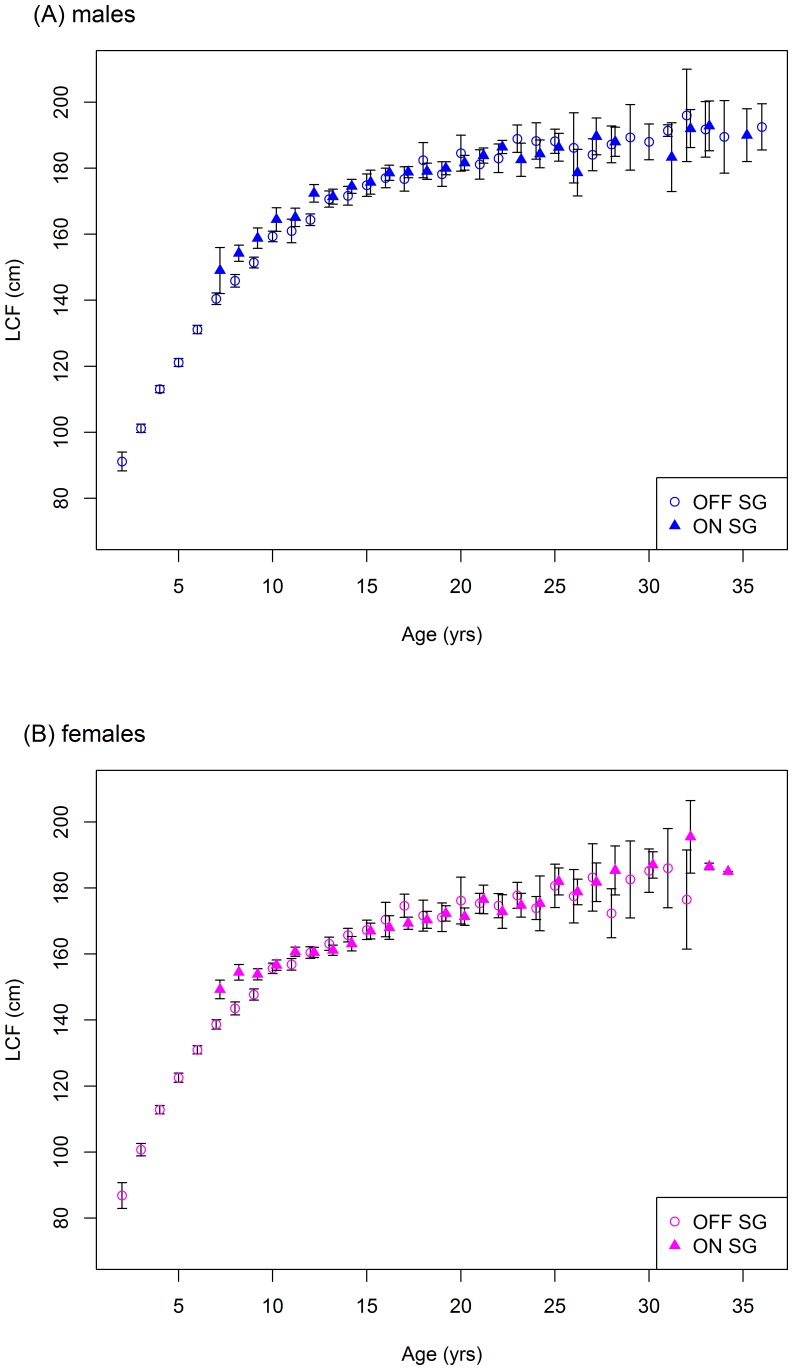
Mean length-at-age ± 2SE for fish caught on and off the spawning ground by sex. SG  =  spawning ground.

Mean length-at-age for males on the spawning grounds is significantly greater than that of females beyond age 10 (Fig. 8). VB growth models fit to the data suggest males grow at a faster rate than females, but that females have a higher asymptotic length and, thus, ‘catch up’ in size by age 35 (Fig. 9). The higher asymptotic length for females may be due to small sample sizes beyond age 25 (see Discussion). Comparing fish caught on and off the spawning grounds show that the mean length-at-age of fish aged 8–10 years is significantly greater on the spawning ground, for both males and females (Fig. 10).

## Discussion

Our results clearly show that the size and age distribution of SBT caught in the Indonesian tropical tuna longline fishery on the SBT spawning ground has undergone substantial changes over the past two decades. These changes appear to be irrespective of changes in fishing practices for which we have information, such as fishing depth (BE index) or month of capture. The catch monitoring showed that there has been a decrease in the relative abundance of larger/older SBT since the early-2000s. It was important, however, to identify fish in the size monitoring data that were caught south of the spawning ground so that these could be excluded from the analyses. SBT catches landed by the company identified as having operated in this south zone contained a greater proportion of small fish (140–160 cm *FL*) compared to the other companies in the 2005 to 2007 seasons and to a lesser extent in 2004. This is consistent with historic Japanese catch data which showed that the mode of SBT caught on the spawning (‘Oka’) ground at 10–20°S was higher than for the staging (‘Oki’) ground to the south at 20–35°S [21]. Although the maturity status of small fish caught in the southern zone was unknown, it is possible that a proportion were immature and undertaking trial migrations towards the spawning ground. Biological sampling and histological analysis of gonads is required to confirm the maturity status of the smaller ‘southern’ fish.

Changes in the size of SBT caught on the spawning ground have been reported previously. The average size of SBT caught by Japanese longliners increased steadily between the early-1970s (161.0 cm *FL*) and early-1990s (169.9 cm *FL*), and it was suggested that the increase was due to a reduction in the number of small SBT in the spawning population or an increase in growth rates over time [13]. In the late-1990s, the mean size of SBT caught on the spawning ground during longline surveys by Japan was lower than that caught by Indonesia, and these differences were explained by different fishing depths targeted by the vessels [11]. The decrease in the mean size (and age) of SBT caught by Indonesia since the early 2000s appears to be independent of changes in fishing depth, but should be considered in relation to effort within the fishery to determine if the change was the result of an increase in number of small/young fish or a decrease in the larger/old fish in the catch. Up until 1997, it was possible to determine catch per unit effort (CPUE) for most Indonesian vessels because it was known how many days vessels had fished and the number of hooks used because the crew were interviewed when they landed their catch. Carrier boats were first noted in the fishery in 1997 when four were registered as using Benoa [22]. This information on the number of sets and the number of hooks used per set was lost as carrier boats became increasingly dominant in the fishery and crews of individual boats could not be questioned as they did not land their catch. Despite the lack of CPUE information, the decrease in mean length and age of SBT observed in the current monitoring data appears to be due, in part, to pulses of higher than average abundance of young fish entering the fishery in the early- and mid-2000s. The absence of a decline in the mean age of fish ≥20 years in the early 2000s suggests that the shift in the age distribution of SBT caught was unlikely to be due to a decrease in the catch of very old fish. The slight decrease in mean age of fish ≥20 years in 2008–2011 may be attributed to the first pulse of recruits entering the 20+ age class in these recent years. The two clear pulses of recruitment in the age frequency data were not apparent in the length frequency time series. This can be explained by the substantial variability in length-at-age of adult SBT obscuring these cohorts in the length frequency data [2], [12].

It is possible that the observed increase in the relative number of smaller/younger fish in catch since the early 2000s occurred as a result of increased juvenile survival following the introduction of quotas in the mid-1980s, leading to more fish surviving though to the spawning age. Quotas have reduced the total catch of SBT from around 40,000 tonnes in the early-1980s to around 10,000 tonnes since 2006 [23]. Importantly, the introduction of quotas significantly reduced the number of juveniles (aged 2–4 years) being caught in surface fisheries around Australia from a peak of just over 21,000 tonnes in 1982 to around 5,000 tonnes since 1990, allowing them to escape to the high-seas longline fisheries. However, the pulse of fish entering the spawning population as say 12 years-olds in 2003 would have been 2–4 year-olds in 1993–1995, after quotas had taken effect, suggesting that both natural variability in recruitment strength and the effect of fishing shape the age structure of the SBT spawning population. Recruitment of young SBT to the New Zealand longline charter fishery is evident in the early-2000s and more recently in 2008 [24]. The later pulse, aged 6–8 years in 2012, may become evident as the beginning of a new pulse of increased abundance in the spawning stock at age 9 years in 2014.

The catch of SBT by Indonesia' longline fishery was dominated by females in the smallest length classes (145–165 cm *FL*), while the catch of SBT by Japanese longliners on feeding grounds in the southern oceans were close to 1∶1 for these length classes [12]. It could be suggested that the determination of sex may not be accurate for SBT in Indonesia since it was done using a small sample of remnant gonad tissue left in the visceral cavity when the fish is landed. Although this method is crude and there is potential for error, the length-at-age data indicated clear sexual dimorphism in growth suggesting that sex has been correctly identified in most cases. The shift to male dominance in the current study occurred at approximately the same length as observed in [12], and the trend of increasing male dominance with size was also very similar. Sex ratios reported for other tuna species are generally 1∶1 with a similar prevalence of males in the larger length classes [25]. The male dominance in large length classes for SBT may be due to the observed sexual dimorphism in growth, as suggested for albacore tuna (*Thunnus alalunga*) in the South Pacific Ocean [26].

The reason for the predominance of females in length classes <170 cm is less clear but may reflect sexual differences in vulnerability or availability on the spawning ground. Females may be more catchable than males if they are more likely to be feeding or actively spawning (i.e., spending a greater proportion of time near the surface targeted by the shallow-setting Indonesian longliners) compared to males of a similar size. Unfortunately, data on feeding, depth partitioning, residency and spawning behaviour are not available for SBT by sex, although females are known to be capable of spawning daily [4]. Regardless of the cause of the bias in sex ratio, a disproportional harvest of females is likely to have implications for population egg production estimates, modeling stock dynamics and fisheries management. A higher catch rate of females by Indonesia over time could ultimately lead to a decrease in the abundance of females in the spawning population, and a subsequent decline in reproductive potential.

The growth rate of male SBT on the spawning ground was found to be greater than that of females, supporting results in Lin and Tzeng [27]. However, unlike Lin and Tzeng [27], our results show a higher asymptotic length for females. This could be due to small sample sizes at the oldest age, because we did not find it to be true when we refit VB models to all of the data, regardless of sexing method (in which case asymptotic length was estimated to be equal for males and females). Sexual dimorphism in the growth of other tuna species has also been found (e.g. albacore [28]). Mean length-at-age of fish aged 8–10 years, both males and females, was greater on the spawning ground than off. This is consistent with [29] which showed that size is a factor in the early maturation of SBT for both sexes.

The observed changes in the length and age structure of the catches since the mid-1990s may not reflect a reduction in the per capita reproductive potential of the spawning stock. Although there has been a decrease in the mean size and age of adults caught by Indonesia on the spawning ground, the change in demographics may be due to an increased abundance of young fish recruiting to the fishery rather than a substantial truncation of the length and age distribution due to fishing mortality. The first pulse of recruits that appeared in the catch in the early 2000s are now reaching ages of ∼20 years and their higher batch fecundity relative to younger females [4] may mean that per capita annual egg production could increase over time.

The size- and age-based metrics presented here are essential to understanding the impact of the Indonesian fishery on the SBT spawning population and form part of a suite of indicators on the status of the SBT stock [30]. The age estimates are used to estimate catch-at-age for the Indonesian fishery which is an important input to the stock assessment models providing information on the adult component of the population and changes over time [31]. These data are also used in the CCSBT operating model which has been used for Management Strategy Evaluation of the adopted SBT management procedure. The management procedure is used to set the global TAC of SBT [32]. The continued collection of the time series of data will be vital for monitoring the rebuilding of the stock and the international management of the species. The monitoring program and these data are also an important component of a new ground breaking method for estimating the abundance of the spawning population of SBT using modern genetic techniques to identify parent-offspring-pairs – the “close-kin” method [33]. However, uncertainties remain in estimating the reproductive potential of SBT. Data is required to accurately estimate the maturity schedule for SBT, understand spawning migrations and reproductive behavior, as well as determine residency and selectivity on the spawning ground.
